# The African Journal Partnership Program's guidance on the use of AI in scholarly publishing

**DOI:** 10.4314/gmj.v58i1.1

**Published:** 2024-03

**Authors:** Caradee Y Wright, Margaret Lartey, Kenza Khomsi, Frederico Peres, Daniel Yilma, James Kigera, Annette Flanagin, Ahia Gbakima, David Ofori-Adjei, Ernest Sumaili Kiswaya, Siaka Sidibé, Adégné Togo, Adamson S Muula

**Affiliations:** Chief Specialist Scientist, South African Medical Research Council and Deputy Editor, Journal of Health and Pollution; Department of Medicine & Therapeutics, University of Ghana Medical School and Deputy Editor-in-Chief, Ghana Medical Journal; Senior Researcher, General Directorate of Meteorology, Morocco and Deputy Editor, Journal of Health and Pollution; Senior Researcher, Sergio Arouca National School of Public Health (ENSP/FIOCRUZ, Brazil) and Deputy Editor, Journal of Health and Pollution; Department of Internal Medicine, Jimma University and Editor-in-Chief of Ethiopian Journal of Health Science; Faculty of Health Sciences, University of Nairobi and Editor in Chief, Annals of African Surgery; Executive Managing Editor and Vice President, Editorial Operations, JAMA and the JAMA Network; Co-Director, African Journal Partnership Program; Editor in Chief, Sierra Leone Journal of Biomedical Research; Editor in Chief, Ghana Medical; Co-Director, African Journal Partnership Program; Deputy editor in chief, Annales Africaines de Médecine, University of Kinshasa, PO BOX 834 Kinshasa, Democratic Republic of the Congo; Editor in Chief, Mali Medical; Faculty of Medicine and Dental Surgery Bamako - USTTB, Editor manager Mali Medical; Editor in Chief, Malawi Medical Journal, Blantyre, Malawi & Department of Community and Environmental Health, Kamuzu University of Health Sciences, Malawi

## Introduction

The rapid introduction and evolution of artificial intelligence (AI), machine learning (ML), natural language processing (NLP), and large language models (LLMs) combined with the emergence of text-generating chatbots have ushered in a transformative era in scholarly publishing. See the Box for common terms and definitions. These technological advancements have the potential to streamline the research and publishing process, from automated content generation and language editing to improved content recommendations and data mining (Table 1). While these innovations offer numerous benefits, they also present scholarly publishing with a range of critical issues that must be addressed.^1^ The use of LLMs and textgenerating chatbots can inadvertently introduce bias, inaccuracies, and ethical concerns into scholarly content, requiring vigilant oversight to ensure the integrity and quality of published research and other content. In addition, the rapid pace of technological advancement demands that the scholarly publishing community establish guidance and best practices for the responsible use of AI in research and publication.

**Table 1:** A selection of trends and initiatives around the use of AI and LLM in scholarly publishing

Trend	Description
Text analysis and data mining	AI and natural language processing (NLP) techniques are used to extract insights from published literature. Researchers use these tools to identify trends, discover relationships between papers and automate systematic reviews.
Content generation	AI tools can be used for content generation, assisting authors in drafting content such as abstracts and summaries, as well as metadata.
Automated author services	AI-based services include language editing, figure preparation, citation, reference formatting, and other technical aspects of manuscript preparation.
Plagiarism detection	Authors use plagiarism detection software to ensure originality and avoid unintentional plagiarism before submission. AI-based plagiarism tools can be used to identify instances of academic misconduct. Authors must mention use of AI since this is a part of plagiarism when left uncited.
AI-enhanced peer review	AI can help identify potential reviewers, check for plagiarism and assist in assessing the quality and validity of research submissions.
Pre-print screening	Manuscripts undergo pre-print screening for initial assessment before formal publishing. AI-powered systems can screen pre-prints and identify potential issues such as ethics concerns, misinformation or research misconduct thereby helping to maintain the quality of scholarly content in pre-print servers.
AI-driven content recommendation	After publication, AI algorithms can assist in recommending relevant articles to readers, thereby increasing the discoverability of scholarly content.
Enhanced accessibility	AI is being used to improve access to academic resources and enhance their usability for a broader audience. It can be used for automated transcription services and alternate text generation for images to assist people with disabilities.
Open access and AI	Open access publishing initiatives are using AI to increase the accessibility of research content making it widely available to the global research community.

While developing such guidance, important principles should be considered such as transparency, responsibility and accountability to ensure that the use of AI adheres to academic standards. Issues around data privacy, authorship attribution and accountability, intellectual property rights, and plagiarism detection all need careful consideration to safeguard the integrity and trustworthiness of research and publication.

The use of AI and LLMs in scholarly publishing is important to promote equity, address specific challenges and opportunities, and empower researchers and publishers to leverage such technologies while ensuring the responsible, inclusive and ethical dissemination of knowledge. Access to advanced AI technologies and LLMs is not uniform across the world. Researchers and clinicians in low- and middle-income countries face a digital divide. Ensuring access to these technologies in scholarly publishing is crucial to prevent further disparities in knowledge creation and dissemination. Although not unique to Africa, there are several challenging issues to address via guidance on the use of AI and LLMs in scholarly publishing in African journals. Adequate data protection measures and best practices are critical to ensure data security. Guidance on how to protect sensitive data is critical, particularly in Africa, where data privacy regulations vary. Also, issues related to intellectual property, plagiarism, and the ownership of AI-generated content should be considered to protect the interests of researchers and institutions.

In Brazil, a recent study^2^ conducted based on an exploratory content analysis raised some important questions on the implications of AI use in academic writing. It showed that AI technologies that generate texts in natural language, such as ChatGPT, are quite developed and increasingly accessible. These tools are becoming popular particularly among graduate students and young faculty for immediately and intuitively generating texts that are supposedly original texts. These trends are associated with the strong pressure to meet increasing academic productivity targets and result in an intensification of plagiarism cases, even when not detected by the most popular anti-plagiarism tools; thereby posing new challenges to editorial groups^3^,^4^ and academic institutions, regarding the need to identify and curb AI-induced academic misconduct. Editors and reviewers will mostly not be able to disentangle what is human-generated or AI-generated knowledge, as the resulting text in a manuscript will often be the mixed result of both. This can bring challenges. For example, some authors, motivated by the professional incentives related to publishing articles, might be enticed to produce large amounts of largely AI-generated content, not all of which may be accurate, thereby potentially overwhelming editors, editorial boards, and reviewers with fact-checking.

Given these new opportunities and challenges, several journals and professional societies of editors have published guidance on AI in scholarly publishing.^5^-^9^ In light of these developments, the African Journal Partnership Program (AJPP) deemed it prudent to develop guidance on the use of AI, NLPs and LLMs in scholarly publishing in their journals. AJPP editors and colleagues reviewed the Committee on Publication Ethics (COPE) guidance on authorship and AI tools^6^, guidance from the World Association of Medical Editors (WAME)^7^ and the International Committee of Medical Journal Editors (ICMJE)^8^ as well as journals or publishers such as the JAMA Network journals^5^ while preparing this guidance for AJPP journals, authors, and peer reviewers. Importantly, AI and AI-assisted technologies should only be used to improve readability and language of the work, and possibly as a ‘brain-storm partner’, and not be used to carry out the work of the researcher(s) such as producing scientific insights, analyzing and interpreting data, or drawing scientific conclusions.

### For authors

The use of AI tools for manuscript preparation is permitted, however, authors remain ultimately answerable and accountable for all content in the manuscript; and authors should be entirely transparent on what AI tools they used and how they used them. Thus, authors should follow these recommendations:

- AI tools must not be listed as authors because they do not meet authorship criteria and cannot be accountable for a published article.
- Authors must disclose to journals at the time of manuscript submission if AI-assisted technologies (such as LLMs, text-generating chatbots, or image creators) were used to produce any of the content in the submitted work. This information can be included in the cover letter; some journals may also have a question about this in the online manuscript submission system.
- Authors must also provide information in the manuscript on which AI tool was used, how it was used, which version was used and the date on which it was used.
  ◦ Authors must report fully on the use of AI to create, edit, or review any content or to assist with those tasks in the Acknowledgment section (name of AI tool, version number, dates of use, prompts entered, and what was done). As much detailed as possible should be provided, such as which sections of the manuscript or other content contain AI generated content, any and if any ideas were generated by AI, these should be described.
  ◦ If use or testing of AI tools, models, or interventions are the focus of a study, a complete description should be provided in the methods or similar section of the manuscript (including name of AI tool, version number, dates used, what was done, and how any potential biases were identified and managed).
- Authors are responsible for verifying the accuracy and appropriateness of any AI generated outputs.
- Citation of AI-generated content as a primary source of information or content is unacceptable.
- Authors must check translation accuracy and grammar correction suggested by AI tools.

### For peer reviewers

Peer reviewers should be aware that one of the main trusts of peer review is confidentiality. Using AI tools may compromise this trust as information on the internet is not confidential. Hence, uploading any manuscript or part thereof into an AI tool may violate the confidential nature of peer review.

- Peer reviewers must not enter any information from a submitted manuscript into an AI model / LLM.

### For editors

Editors continue to hold authors accountable for producing unbiased quality content, regardless of how this content is generated.

- Editors are responsible for sharing standards and policies for appropriate and transparent use of AI with authors and peer reviewers.
- The role of editors includes implementing and managing AI-like tools to help improve the efficiency of the manuscript submission and editorial and peer review processes (e.g., checking for submitted manuscripts similarity with other content or plagiarism and matching peer reviewers with manuscripts via key words), incorporating these tools effectively into the editorial process.
- Editors should not base editorial decisions solely on assessments generated by AI tools (e.g., software used to attempt to identify if content may have been generated by AI or to predict acceptability or post-publication performance of submitted manuscripts).
- Editors support authors in complying with guidelines for proper AI utilization and stay informed about advancements in AI technology to guide and facilitate the effective and ethical integration of AI in scholarly publishing.
- Editors should clearly communicate policies on the use of AI in author and reviewer guidelines.

In summary, AI tools in scholarly publishing will become increasingly relevant as knowledge about and use of AI grow. Recommendations will be in a state of flux as editors and publishers review developments and implement policies and processes. Journal editors and publishers need to be acutely aware of this responsibility. They should inform and guide authors and peer reviewers of best practices, build capacity of editors editorial staff to use AI-like tools effectively within the manuscript submission and editorial processes, and develop policies to prevent and manage inappropriate use. The current recommendations, which are in line with international standards, will need careful constant review as circumstances change.

## Defining Terms

**Artificial Intelligence (AI)** is a broad term encompassing computer systems, algorithms and technologies that exhibit behavior or perform tasks that can be considered ‘smart’. These could include making decisions, recognizing and translating speech, and different types of visual perception.^6^

**Chatbot** is a “computer program designed to simulate conversation with human users, especially over the internet”.^9^

**Large language model (LLM)** “learns the probabilities of occurrence of sequences of words from a corpus of text, whose probabilities are learned using textual corpora with trillions of words such that the resulting model has billions of parameters”.^10^

**Machine learning (ML)** is a subset of AI and consists of algorithms that detect patterns based on a pool of training data, and use them to predict, detect and make decisions.^6^

**Natural language processing (NLP)** is the application of computational techniques to the analysis and synthesis of natural language and speech.^6^
